# Risks associated with antiretroviral treatment for human immunodeficiency virus (HIV): qualitative analysis of social media data and health state utility valuation

**DOI:** 10.1007/s11136-017-1519-3

**Published:** 2017-03-24

**Authors:** Louis S. Matza, Karen C. Chung, Katherine J. Kim, Trena M. Paulus, Evan W. Davies, Katie D. Stewart, Grace A. McComsey, Marshall W. Fordyce

**Affiliations:** 1 Outcomes Research, Evidera, 7101 Wisconsin Avenue, Suite 1400, Bethesda, MD 20814 USA; 2 Jazz Pharmaceuticals, Palo Alto, CA USA; 3 Genentech Pharmaceuticals, San Francisco, CA USA; 4 University of Georgia, Athens, GA USA; 5 Actelion Pharmaceuticals, Allschwil, Switzerland; 6 Case Western Reserve University, Cleveland, OH USA; 7 CDF Therapeutics, Inc., Burlingame, CA USA

**Keywords:** Human immunodeficiency virus, Acquired immune deficiency syndrome, Antiretroviral, Treatment risk, Utility, Time trade-off

## Abstract

**Purpose:**

Despite benefits of antiretroviral therapies (ART), people with HIV infection have increased risk of cardiovascular disease, kidney disease, and low bone mineral density. Some ARTs increase risk of these events. The purpose of this study was to examine patients’ perspectives of these risks and estimate health state utilities associated with these risks for use in cost-utility models.

**Methods:**

Qualitative thematic analysis was conducted to examine messages posted to the POZ/AIDSmeds Internet community forums, focusing on bone, kidney, and cardiovascular side effects and risks of HIV/AIDS medications. Then, health state vignettes were drafted based on this qualitative analysis, literature review, and clinician interviews. The health states (representing HIV, plus treatment-related risks) were valued in time trade-off interviews with general population participants in the UK.

**Results:**

Qualitative analysis of the Internet forums documented patient concerns about ART risks, as well as treatment decisions made because of these risks. A total of 208 participants completed utility interviews (51.4% female; mean age 44.6 years). The mean utility of the HIV health state (virologically suppressed, treated with ART) was 0.86. Adding a description of risk resulted in statistically significant disutility (i.e., utility decreases): renal risk (disutility = −0.02), bone risk (−0.03), and myocardial infarction risk (−0.05).

**Conclusions:**

Patient concerns and treatment decisions were documented via qualitative analysis of Internet forum discussions, and the impact of these concerns was quantified in terms of health state utilities. The resulting disutilities may be useful for differentiating among ARTs in economic modeling of treatment for patients with HIV.

## Introduction

Human immunodeficiency virus (HIV), which causes acquired immunodeficiency syndrome (AIDS), continues to be a major health concern worldwide [1–3]. Treatment with antiretroviral therapies (ARTs) has been shown to be effective in controlling the virus, preventing symptoms, increasing life expectancy, and reducing risk of transmitting the virus to others [4–8]. However, there is growing awareness of risks associated with commonly used ARTs. For example, abacavir, which is a key component of several commonly used treatment regimens, has been associated with cardiovascular risk, possibly including elevated risk of myocardial infarction for some patient groups [9–11]. Tenofovir disoproxil fumarate, another commonly used ART, has been associated with risk of bone mineral density loss [12–14] and renal dysfunction [15–17]. Patients’ concerns about these risks could have an impact on quality of life and treatment preference, even among patients who have not experienced the actual side effects or medical events.

Cost-utility analyses (CUAs) are frequently conducted to estimate and compare the value of ARTs [18–24], and results of these models are used to inform decisions regarding allocation of healthcare resources. In CUAs, treatments are assessed in terms of cost per quality-adjusted life year (QALY) gained [25]. QALYs are calculated with health state utilities, which represent the strength of preference for a given health state on a scale anchored to 0 (dead) and 1 (full health) [26, 27]. Although utility values are available for HIV [28–38], no previous studies have estimated the disutility (i.e., utility decrease) of living with the risks of ARTs, including risk of cardiovascular disease, bone mineral density loss, and renal dysfunction.

Therefore, the two goals of this study were to (1) explore patients’ perspectives of the risks of ART and (2) estimate health state utilities associated with these risks so that the values may be used in CUAs. This study was conducted in two phases, each corresponding to one of the two study goals. First, to better understand patients’ experiences of living with ART associated risks, a qualitative thematic analysis was conducted of public Internet forums in which patients with HIV describe their experiences and discuss treatment options. Social media data, such as these Internet forums, can be a rich source of information from a broad range of patients, providing an efficient way to explore the patient perspective [39].

Second, the qualitative data gathered in Phase 1 were used to support the development of health state vignettes that were rated in a time trade-off (TTO) utility valuation. This valuation study was designed to identify the disutility of living with risks associated with ART. Whereas previous utility studies have estimated disutility associated with experiencing treatment-related adverse events, the current study is the first to examine whether there may be a disutility associated with elevated risk of adverse events, regardless of whether the event occurs.

Although some health technology assessment guidelines such as those issued by the UK National Institute for Health and Care Excellence (2013) have expressed a preference for utilities derived from generic preference-based measures, these generic instruments are not likely to be sensitive to the impact of living with risk [40]. Therefore, the current study used a vignette-based approach, which is well-suited for isolating the impact of specific treatment-related attributes on utility. In this case, the disutility of risk was estimated by valuing HIV health states differing only in the presence of the risks. The utility difference between two otherwise identical health states, one without the risk and one with the risk, can be fully attributed to the risk.

## Phase 1: methods

### Phase 1 overview: thematic analysis of HIV/AIDS discussion forum data

Phase 1 involved a qualitative thematic analysis [41–43] of messages posted to the POZ/AIDSmeds community forums (conducted by co-author TP with input from LM and KC). Posts were analyzed for discussion of bone, renal, and cardiovascular risk associated with ART to support the development of health state descriptions valued in Phase 2.

### Data source

The asynchronous, text-based public discussion forums sponsored by POZ Magazine were selected as the data source because of the active community, robust membership, and high level of interactivity. The forums are described by POZ as “a round-the-clock discussion area for people with HIV/AIDS, their friends/family/caregivers, and others concerned about HIV/AIDS,” all of whom are considered “members” of the forum for the purposes of this study. The forums began in 2006 and had over 23,000 members by August 2014 when the data were retrieved. All messages are publicly visible on the Internet.

There are 18 forums organized by topics including HIV transmission, living with HIV, AIDS activism, and treatment and side effects. For the current analysis, all posts made to two forums between January 2008 and August 2014 were retrieved: (1) the English version of “Living with HIV” which is the largest forum (191,194 posts in 12,457 topics) and (2) “Questions about treatment and side effects” (32,310 posts in 3710 topics).

### Data extraction and analysis

The qualitative data analysis software ATLAS.ti version 7.0 was used to search the data for terms related to side effects and long-term risks of HIV/AIDS medications: cardio*, heart*, angina, myocardi*, stroke*, kidney*, renal*, bone*, osteo*, fracture*, adverse*, side effect*, and risk*. All posts containing these terms were auto-coded, resulting in 4122 coded data segments known in ATLAS.ti as “quotations.” These quotations were reviewed for relevance, and duplicate and irrelevant quotations were deleted. Quotation size was adjusted to capture the relevant section of each post, resulting in one quotation per post.

**Fig. 1:**
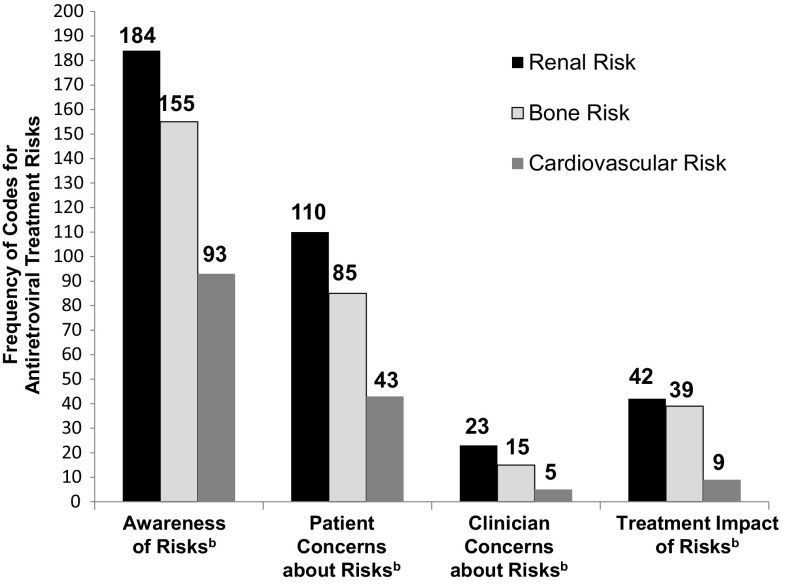
Qualitative analysis of POZ/AIDSmeds community forums: frequency of codes indicating impact of risks of antiretroviral treatment. ^a^There are 18 separate POZ/AIDSmeds forums organized by topics. For the current analysis, all posts made to two forums between January 2008 and August 2014 were examined: (1) the English version of “Living with HIV” and (2) “Questions about treatment and side effects.” ^b^Four risk impact codes were used to categorize text of posts in online discussion forums using Atlas.ti version 7 qualitative data analysis software. Discussion of risk was further categorized into three content areas: bone risk, renal risk, and cardiovascular risk. *Awareness* Statements indicating awareness of risks of ART; *Patient concerns* Statements indicating patient concerns or emotions related to risks of ART (e.g., worry); *Clinician concerns* Statements in which a patient mentions that his/her healthcare provider was concerned about risks of ART; *Treatment impact* Statements indicating that a pharmaceutical treatment was changed due to an actual side effect of ART or a known risk of ART; *ART* antiretroviral treatment

Quotations were analyzed as follows. Initial data search terms were consolidated into three major risk codes: bone (includes osteo and fracture), kidney (includes renal), and heart (includes cardio, stroke, myocardi, and angina). Posts were then coded according to four types of risk impact in order to document the extent to which patients and clinicians are concerned about these risks (defined in footnote of Fig. 1): (1) awareness of risk, (2) patient concerns about risk, (3) clinician concerns about risk, and (4) treatment impact resulting from risk. It was possible for a single quotation to receive multiple codes. Names of ARTs were also coded.

## Phase 1: results

### Summary of results

A total of 614 relevant posts were identified across 337 different discussion topics (threads). The discussion topics that mentioned bone, renal, and cardiovascular risks most frequently were titled “Med other than Truvada or Epzicom,” “HIV MEDS and Osteoporosis,” “Tenofovir associated with significant kidney damage,” “Stopping Truvada because of osteoporosis,” “Isentress/Truvada side effects,” “Starting Viramune (Nevirapine) + Kivexa (Epzicom = 3TC/abacavir),” “Kidneyfailure (GFR39),” “Question about switching out Truvada,” “Truvada and Borderline osteoporosis…Epzicom substitute?,” and “Atripla and kidney disease.” Some discussion topics lasted only a few days, while others lasted for years, such as the “Starting Viramune” topic that lasted for nearly four years and had 156 posts.

As is typical in Internet discussion forums, many more people appeared to read posts than contribute posts. For example, while only nine posts were made to the “Stopping Truvada because of osteoporosis” topic, the posts were viewed 1355 times. Similarly, 41 posts were made to the thread titled “Tenofovir assoicated [sic] with significant kidney damage,” which received 9226 page views. A total of 205 members contributed these 614 posts to the forums. Thirty-four members made more than three posts related to the three risk categories, while 10 members made more than 10 posts of interest.

A total of 31 drug names were mentioned in the posts, 24 of which were mentioned in more than three posts. Truvada was mentioned most frequently (232 posts), followed by tenofovir (118), abacavir (63), Atripla (60), Viread (55), Epzicom/Kivexa (51), Stribild (45), atazanavir (38), Norvir (29), Isentress (26), efavirenz (23), and Kaletra (23). Bone and renal risks were discussed most frequently in conjunction with tenofovir disoproxil fumarate and Truvada, while cardiovascular risks were discussed most frequently in conjunction with Abacavir.

### Qualitative coding results

Qualitative coding identified statements mentioning one or more of the three risk categories: bone (*n* = 270), renal (*n* = 336), and/or cardiovascular (*n* = 142). Of the 614 posts, 352 conveyed awareness of the risk, 191 indicated patient concern, 38 conveyed patients’ reports of clinician concern, and 77 reported a change in treatment because of the risk. These codes are not mutually exclusive. For example, some quotations expressed both clinician concern and treatment impact, and these quotations were double-coded.

Within each of the three risk categories, coding revealed all four types of risk impact (see Fig. 1 for frequencies of codes). Examples of quotations for each type of risk are presented in Tables 1 (bone), 2 (renal), and 3 (cardiovascular). Members often shared their treatment plans, reported their clinical condition (e.g., decreasing bone density), expressed concern, and requested feedback on possible treatment changes. Furthermore, patients commonly reported a change in pharmaceutical treatment due to either actual side effects or risk of side effects.

**Table 1:** Qualitative analysis of POZ/AIDSmeds community forums^a^: examples of quotations coded for impact of bone risks associated with antiretroviral treatment

Codes for impact of bone risks	Quotations from POZ/AIDSmeds community forums
Awareness	“HIV meds, especially Viread (in Atripla and Truvada) can cause bone loss. I take Vitamin D/calcium also about 3 times a week” “A couple parts of my body are borderline osteoporosis. I am starting to get pain in my right hip and lower back. I am understanding Truvada is most likely the cause” “So I went to the doc today about these bone issues...I’m having...He says the Viread in Atripla can cause bone issues, but usually only when someone has been taking it for a long while” “I looked at some online studies that show that tenofovir is to blame for possible bone issues; even my doctor confessed this”
Patient concerns	“My concern with Truvada is bone related. I’ve had osteopenia since 2003. No one told me (before or after the bone loss) that Tenofovir could cause bone loss…Do you know whether going off Tenofovir would potentially improve my osteopenia or at least prevent it from getting worse?”
Clinician concerns	“A couple parts of my body are borderline osteoporosis. I am starting to get pain in my right hip and lower back. I am understanding Truvada is most likely the cause. My doctor suggested a change to Epzicom” “I was tested for bone density and the results were I have a certain degree ofosteopenia (loss of bone density). My doc said it could be due to the tenofovir contained in Truvada, but said also that many HIV + people develop osteopenia even when taking other meds, so she appointed me for a new bone density test by the end of this year to see whether it has progressed” “I started on Atripla…and my bone density test shows some bone thinning…My PCP is thinking it maybe from the Atripla or my parathyroid. My ID doctor said, Atripla can in a few cases cause this problem. So, he has now started me on Norvir, Epzicon and Reyataz. He doesn’t like one of the ingredients in Truvada long term due to bone density stuff, think that was his rationale for not using a once a day pill”
Treatment impact	“…diagnosed with osteporosis in feb after 5 years on meds...now I successfully switched to Epzicom instead of Truvada. I have dropped tenofovir from my combo because of long-term concerns about bone health” “I had the same problem 4 years ago. My doc changed my meds from Truvada + Reyataz to Truvada + Intellence (Etravirine, an NNRTI). And, a year later, to Kivexa + Intellence. It seemed to partially stop bone density loss. But I haven’t recovered the density I lost in those first years on treatment” “I just switched from Truvada Reyatz + Norvir to Truvada Intellence this week...I have developed osteopenia, maybe due to combined effect of Truvada and Reyataz on calcium metabolism”

^a^There are 18 separate POZ/AIDSmeds forums organized by topics. For the current analysis, all posts made to two forums between January 2008 and August 2014 were examined: (1) the English version of “Living with HIV” and (2) “Questions about treatment and side effects”

**Table 2:** Qualitative analysis of POZ/AIDSmeds community forums^a^: examples of quotations coded for impact of renal risks associated with antiretroviral treatment

Codes for impact of renal risks	Quotations from POZ/AIDSmeds community forums
Awareness	“Regarding Stribild and the kidney issue. Yes it is an issue. How big of an issue is still a question. Because Stribild is so new there isn’t a lot of data. All we know is that it does affect your kidneys because it causes certain kidney tests to be elevated. Combine that with Truvada’s effect on your kidneys and it’s a double whammy. Some doctors are hesitant in prescribing it because there are still some unanswered questions (including mine)” “I just started on stribild. I really wanted it because of all the good things I’ve read. I’m getting kidney stuff checked frequently and so far haven’t had any reason to believe my kidneys aren’t holding up just fine” “I hear that Atripla can cause liver and kidney damage long-term and can cause other organ damage. Has anyone experienced any severity of this?”
Patient concerns	“The last week or so I have experienced lower back pain? I know Truvada can be associated with kidney problems, but I won’t get my first lab test for another 3 weeks so I won’t know if it is my kidneys causing the pain. Should I be worried?” “I’m not thrilled with Truvada since I have concerns about long-term kidney issues that it can possibly cause” “I take Stribild…The only thing I’m concerned about is the effects it’ll have on my kidneys. I have been doing clinical at an HIV clinic for school and I’m astounded at the amount of people that have renal disease requiring dialysis”
Clinician concerns	“I wanted to start Stribild and my doc said if I really insisted on it, she would give it to me. However she did mention the long-term effects that weren’t known, and I also have a history of kidney stones so she said it’d be a possible increased risk for more kidney issues” “So here’s my question: my nurse, whom I respect and appreciate dearly…She doesn’t like the Truvada very much, saying it’s prone to cause renal problems” “Because of my kidney/kidney stones/always having RBC in my UA, she was leery of Stribild. She said Stribild is a good drug but can really be damaging to your kidneys. She said, though my kidney function is fine right now, there’s no long-term studies to show how it will effect healthy kidneys over time and also being as how I’ve had issues already, she didn’t want to chance it”
Treatment impact	“I was taken off Truvada because of low kidney function. The good news is Doc said I might regain function after a bit” “The switch to Epzicom was very uneventful...My kidney numbers are slowly creeping back toward normal—not sure how long it will take for them to fully recover, but I’m happy that I made the change” “I was also on Reyataz and had to stop after some kidney dysfunction due to Reyataz”

^a^There are 18 separate POZ/AIDSmeds forums organized by topics. For the current analysis, all posts made to two forums between January 2008 and August 2014 were examined: (1) the English version of “Living with HIV” and (2) “Questions about treatment and side effects”

**Table 3:** Qualitative analysis of POZ/AIDSmeds community forums^a^: examples of quotations coded for impact of cardiovascular risks associated with antiretroviral treatment

Codes for impact of cardiovascular risks	Quotations from POZ/AIDSmeds community forums
Awareness	“I read that Epzicom has moved from ‘preferred’ to ‘alternate status because concerns about possible increased heart attack risk”
Patient concerns	“…the Abacavir added risk to MI, it is indeed a concern of mine. I am not too happy with this additional risk. Therefore I will discuss it with the cardiologist” “Actually my doc has brought up another boosted PI with Isentress, but I am reluctant, because I think the Norvir booster (even at that low dose) is heart toxic…Probably being a little irrational, but still...” “My concern with Epzicom is that I took it for 5 years. I only switched because of abacavir and the studies that showed potential cardio problems”
Clinician concerns	“I would like to switch since the doctors are concerned about heart attack risk with ziagen” Abacavir: “My doctor said he will not prescribe it for me due to the combined facts of my age (48) and I’m a smoker. He said the cardiac risk was too high when there were other meds available to me”
Treatment impact	“My concern with Epzicom is that I took it for 5 years. I only switched because of abacavir and the studies that showed potential cardio problems” “Ziagen (abacavir) is now said to be linked to heart disease. In my case, with a family history of heart attacks, I am changing my regime to Truvada plus something else…” “I switched from Epzicom to Atripla—around 2007—because of general cardiovascular concerns with abacavir—even though I don’t have risk factors for heart disease, except I’m a male and 60” “I switched from Kaletra…I felt Kaletra may have been keeping my HIV under control but it was putting me at risk for stroke”

^a^There are 18 separate POZ/AIDSmeds forums organized by topics. For the current analysis, all posts made to two forums between January 2008 and August 2014 were examined: (1) the English version of “Living with HIV” and (2) “Questions about treatment and side effects”

Overall, the qualitative analysis of social media data revealed that ART-related risks are a concern for some patients and clinicians, and these risks may influence treatment decisions. Based on a substantial number of quotations, it appeared that the risks associated with some ARTs could have a meaningful impact on patient quality of life and treatment preference. Therefore, it was determined that it could be useful to quantify this impact in terms of utilities so that these differences among ARTs could be incorporated into economic modeling of these treatments.

## Phase 2: methods

### Phase 2 overview: health state utilities associated with risks of antiretroviral treatment

Phase 2 was a vignette-based TTO utility study designed to estimate the disutility of living with risks of ART. Health state descriptions were drafted based on literature review, input from clinicians who treat patients with HIV, and the qualitative analysis in Phase 1. Then, the health states were refined based on additional clinician interviews and a pilot study with general population participants in London, UK. Finally, health state utilities were elicited using TTO methods [25] with general population participants in Edinburgh and London, UK. Participants were required to provide written informed consent before completing study procedures, and all procedures and materials were approved by an independent Institutional Review Board (Ethical and Independent Review Services; Study Number 14150-01A).

### Health state development

A clinician interview discussion guide was drafted based on findings from the qualitative analysis in Phase 1 and results of a targeted literature review. This literature review was performed to inform the clinician interview questions and ensure that the health state descriptions were consistent with published research. Literature searches focused on symptoms, impact, and treatment of HIV [7, 8, 44, 45] as well as risks associated with ART, including bone [12–14], renal [15–17], and cardiovascular risks [9–11].

To further inform health state content, telephone interviews were conducted individually with six clinicians (all with MD degrees) including specialists in infectious disease (*n* = 2), HIV (*n* = 2), cardiology (*n* = 1), and nephrology (*n* = 1). First, interviews were conducted with three clinicians to gather information for the first draft of the health states, with questions focusing on patients’ typical experiences with HIV and treatment-related adverse events, including symptoms, impact, and treatment. Clinicians’ descriptions of living with HIV and associated ART risks were generally consistent with each other and consistent with findings from the qualitative analysis of social media data in Phase 1 of the current study. Interviews were also conducted with a nephrologist to inform descriptions of chronic kidney disease (CKD) and a cardiologist to gather information for the post-myocardial infarction description, which was based on a health state used in a previous study [46].

Then, draft health states were sent to three of the original five clinicians and one new clinician. These follow-up discussions focused on ensuring that the descriptions of ART risks were clear, accurate, and not exaggerated beyond what was known based on currently published studies. This process of refining the health states was iterative, and clinicians were interviewed multiple times.

A total of eight health states were drafted. A basic health state (i.e., health state 1) designed to provide context for the three ART risks described a virologically suppressed patient with HIV treated with ART. Three health states included health state 1, plus either bone, renal, or cardiac treatment-related risks. Four health states included health state 1, plus a description of living with actual medical conditions that could arise based on these risks (either CKD or post-myocardial infarction). These four health states were included to ensure that respondents differentiated between living with risk (i.e., health states 2 to 4) and living with the actual medical condition (i.e., health states 5 to 8). To avoid potential bias associated with the disease labels [47], none of the health states explicitly named HIV or AIDS. See Appendix A for full health state text.

### Pilot study

The health states were tested in a pilot study in November and December 2014 with 30 general population participants in London, UK (16 males; mean age 40.3 years; age range 19–67). Each participant valued the health states in a TTO task with several time horizons (e.g., 10 years, 20 years) and trading increments (e.g., 5%, 10%). In TTO procedures, the duration of time in the health state being rated (i.e., the time horizon) varies across studies [48]. In the pilot, the time horizon had no apparent impact on participants’ responses or comprehension of the task. Therefore, it was determined that the subsequent valuation study would use a time horizon of 10 years, which maximizes comparability with many previously published TTO studies including the influential Measurement and Valuation of Health study [49, 50]. Participants generally reported that the health states were clear and comprehensible, although minor formatting and text edits were made based on specific comments.

### Participants

Participants in the pilot and main studies were required to be at least 18 years old; able to understand assessment procedures; able and willing to provide written informed consent; and reside in the UK. Inclusion criteria did not specify clinical characteristics because this study aimed to estimate utilities for CUAs in submissions to health technology assessment agencies, which often prefer that utilities represent general population values [40, 51, 52]. Participants were recruited via newspapers and online advertisements.

### Valuation study data collection and statistical analysis procedures

Utilities for the final health states were elicited in a valuation study in February 2015 (directed by author LM, with interviews conducted by authors LM, KK, ED, and additional interviewers listed in the acknowledgments section). As an introductory task, participants were presented with the basic health state (i.e., health state 1) followed by the remaining health states in random order and asked to rank them in order of preference. Participants then valued the health states in a TTO task with a 10-year time horizon and 6-month (i.e., 5%) trading increments. Participants were offered a choice between living 10 years in the health state being rated or a shorter duration in full health. For each health state, choices were presented in an order that alternated between longer and shorter amounts of time in full health (i.e., 10 years, 0 years, 9.5, 0.5, 9.0, 1.0, 8.5…), and utility scores (u) were calculated based on the point of indecision as the number of years in full health (x) divided by the number of years in the health state being rated (u = x/10 years). Each health state considered better than dead received a utility score on a scale with the anchors of dead (0) and full health (1).

When participants indicated that a health state was worse than dead, the task and scoring procedures were altered as described in previous literature [53, 54]. Participants were offered a choice between immediate death (choice 1) and a 10-year life span (choice 2) beginning with varying amounts of time in the health state being rated, followed by full health for the remainder of the 10-year life span. The resulting negative utility scores may be calculated with one of two possible scoring approaches based on the TTO choice in which the respondent is indifferent between the two alternatives. The first approach yields utilities with a possible range of 0 to −∞, using the formula −x/(10−x) where x is time in full health, and 10 years is the total life span of alternative 2 in the TTO choice (i.e., years in the health state being rated plus subsequent years in full health). These unbounded negative values can skew the distribution of utility estimates for a health state rated as worse than dead by even a small number of respondents. Therefore, the current study used an alternative bounded scoring approach, which is commonly used to avoid highly skewed distributions. The formula is −x/10, where x is the number of years in full health, and 10 is the number of years in the total life span of alternative 2. With this approach, the score range of health states worse than death are limited or “bounded” between 0 and − 1 [53, 54].

Statistical analyses were completed using SAS version 9.2. Continuous variables are summarized in terms of means and standard deviations, and categorical variables are summarized as frequencies and percentages. Disutilities of ART risks and living with related health conditions were calculated by subtracting the utility of health state 1 from each other health state. Demographic subgroups were compared with Chi-square analyses (for categorical variables) and t-tests (for continuous variables). Pairwise t-tests compared health state 1 to every other health state.

## Phase 2: results

### Sample characteristics

A total of 218 participants attended interviews, ten of whom were unable to complete the utility interview procedures (e.g., insufficient comprehension of the health states or TTO procedures). Therefore, the analysis sample included 208 participants (105 Edinburgh; 103 London; 51.4% female; mean age 44.6; age range 18–81). Participant characteristics are summarized in Table 4. The only significant difference between the Edinburgh and London subgroups was that a greater percentage of participants in Edinburgh reported ethnic/racial background as white (92.4 vs. 58.3%; *p* < 0.01). The most commonly reported health conditions were anxiety (14%), depression (12%), bone fracture (8%), arthritis (8%), and diabetes (6%). No participants reported having HIV or AIDS.

**Table 4:** Demographic characteristics

Demographic characteristics	Edinburgh (*N* = 105)	London (*N* = 103)	Total sample (*N* = 208)
Age (Mean, SD)	45.5 (17.1)	43.7 (16.7)	44.6 (16.9)
Gender (*n*, %)			
Male	51 (48.6%)	50 (48.5%)	101 (48.6%)
Female	54 (51.4%)	53 (51.5%)	107 (51.4%)
Ethnic/racial background (*n*, %)			
White	97 (92.4%)	60 (58.3%)	157 (75.5%)
Mixed	4 (3.8%)	8 (7.8%)	12 (5.8%)
Asian	3 (2.9%)	14 (13.6%)	17 (8.2%)
Black	0 (0.0%)	17 (16.5%)	17 (8.2%)
Other^a^	1 (1.0%)	4 (3.9%)	5 (2.4%)
Marital status (*n*, %)			
Single	51 (48.6%)	55 (53.9%)	106 (51.2%)
Married/living with partner	42 (40.0%)	33 (32.4%)	75 (36.2%)
Other	12 (11.4%)	14 (13.7%)	26 (12.6%)
Employment status (*n*, %)			
Full-time work	38 (36.2%)	33 (32.0%)	71 (34.1%)
Part-time work	26 (24.8%)	26 (25.2%)	52 (25.0%)
Other^b^	41 (39.0%)	44 (42.7%)	85 (40.9%)
Education level (*n*, %)			
University degree	43 (41.0%)	42 (40.8%)	85 (40.9%)
No university degree	62 (59.0%)	61 (59.2%)	123 (59.1%)

^a^Other ethnic/racial background includes Arab (*n* = 2), Kurdish (*n* = 2), and South American (*n* = 1)

^b^Other employment status includes homemaker/housewife (*n* = 4), student (*n* = 32), unemployed (*n* = 10), retired (*n* = 32), and disabled (*n* = 4)

### TTO utility values

The mean utility for the basic health state (health state 1) describing a virologically suppressed HIV patient was 0.86 (Table 5). Adding the three ART-related risks in health states 2, 3, and 4 resulted in disutilities of 0.025 for risk of bone problems, 0.018 for risk of renal problems, and 0.049 for risk of cardiovascular problems. Disutilities associated with the medical conditions described in health states 5 to 8 were larger, ranging from 0.057 for living post-myocardial infarction to 0.596 for stage 5 CKD. T-tests comparing the utility of the basic health state to the utility of the other health states found that all differences (i.e., disutilities) were statistically significant (*p* < 0.0001).

**Table 5:** Time trade-off (TTO) utility scores (*N* = 208)

Health states^a^	Mean utility^b^	SD	95% CI	Disutilities: difference from health state 1	
				Mean	SD
1. Basic health state (HIV)	0.862	0.144	0.842–0.881	–	–
Basic health state plus ART risks					
2. Risk of renal problems	0.844	0.149	0.824–0.864	0.018	0.043
3. Risk of bone problems	0.836	0.157	0.815–0.858	0.025	0.078
4. Risk of cardiovascular problems	0.813	0.158	0.792–0.835	0.049	0.084
Basic health state plus other medical conditions					
5. Stage 3 CKD	0.797	0.175	0.773–0.821	0.065	0.102
6. Stage 4 CKD	0.672	0.279	0.634–0.710	0.189	0.232
7. Stage 5 CKD	0.266	0.447	0.205–0.327	0.596	0.440
8. Post-myocardial infarction	0.805	0.165	0.782–0.827	0.057	0.091

*ART* antiretroviral treatment, *CKD* chronic kidney disease

^a^Health states 2 to 8 include the basic health state, plus an additional risk (in health states 2–4) or medical condition (in health states 5 to 8)

^b^TTO scores are on a scale anchored with 0 representing dead and 1 representing full health

T-tests were conducted to compare mean utilities and disutilities across subgroups. There were no statistically significant differences between men and women or between the Edinburgh and London subgroups in mean utilities or disutilities for any of the health states. A comparison of older (>46 years) vs. younger (≤46) respondents, categorized based on a median split, found no differences in utility or disutility for health states 1 to 7. However, younger participants had a significantly lower utility for health state 8 (0.78 vs. 0.83; *p* = 0.02), as well as a significantly greater disutility for this health state (0.07 vs. 0.04; *p* = 0.01) compared to older participants.

Only two of the health states received any negative valuations. Health state 6 was perceived to be worse than dead by four of the 208 respondents, and health state 7 was rated worse than dead by 35 respondents. The other six health states were perceived to be better than dead by all 208 respondents.

## Discussion

While ARTs have transformed the management and progression of HIV, the current study suggests that ARTs which are not associated with increased bone, renal, or cardiovascular risks are likely to be preferred over those that carry these risks. This investigation was facilitated by two methodological innovations. First, this study introduces the use of data from patient Internet forums to support health state utility research. For previous vignette-based utility assessment, health state descriptions have typically been drafted based on literature review, clinician interviews, and occasionally patient interviews. Current findings suggest that data from online discussion forums can add further support for health state vignettes by providing a broad picture of patients’ experiences with relevant disease and treatment attributes. There is growing awareness that social media data can be useful in development of patient-reported outcomes instruments [39]. The current study suggests that data from Internet discussion forums can also be useful for supporting utility research.

The qualitative analysis found that, among patients with HIV and their treating clinicians, there is awareness of the side effects and long-term risks of ARTs for bone, renal, and cardiovascular health. Furthermore, the extracted quotations highlight the potential impact of these risks on emotional well-being, quality of life, and treatment choices. Overall, this qualitative research suggests there is an impact of living with treatment-related risks, even in the absence of actual side effects or medical problems.

A second methodological innovation is the focus on disutility of living with risk, regardless of whether the adverse event or medical condition actually occurs. Previous studies have consistently demonstrated that health state utilities are significantly reduced as a result of bone problems [55, 56], renal disease [57–59], and cardiovascular disease [60, 61]. The current study adds to this research by demonstrating that awareness of elevated risk of these medical conditions, rather than the conditions themselves, may also have a measurable impact on utility. As expected, the disutility of risk (health states 2 to 4) was smaller than the disutility of the medical conditions represented by health states 5 to 8 and quantified in previous research [55–61]. However, the relatively small disutilities associated with ART risks, ranging from 0.018 to 0.049 (Table 5), could still have an impact on the outcomes of a cost-utility model comparing treatments that differ in terms of these risks. In particular, cumulative impact of small differences in utility may be meaningful when modeling long-term treatment of chronic conditions such as HIV.

Although patient Internet discussion forums are a potentially rich source of information, the limitations of online data must be acknowledged. Because members of the online community participate under pseudonyms, it is not possible to know their demographic characteristics or verify their HIV status and treatment experience. Therefore, it is not possible to know the extent to which findings are representative of the broader population of patients with HIV. Patients who communicate online could be different from other patients, and if so, online forum data may represent a potentially biased sample. For example, patients who post in online forums may be more aware of disease and treatment characteristics than other patients. Furthermore, as in many online communities [62], a small number of members of these HIV forums appeared to post frequently, while others actively followed the discussions, but posted less often. Therefore, quotations do not equally represent the entire online community. Future research with a clinic-based sample could further examine the impact of ART risks to replicate findings from the online forums.

Despite limitations, online patient forums are a potentially useful source of information for research in which it is important to understand the patient experience. With these publicly available discussion texts, it is possible to efficiently gain insight into patients’ reactions to disease and treatment. Although anonymity of the discussants limits the ability to verify diagnosis and treatment, this anonymity could also be an advantage. In this anonymous setting, patients may be more comfortable raising sensitive issues or concerns that they may not discuss with clinicians or researchers. Furthermore, because these forums include data from many patients, they provide a way to examine concerns that affect small numbers of patients, but could be important nonetheless. An interview study could fail to detect such concerns that may be important to some patients. Therefore, it is recommended that online patient discussion forums be considered as a potential data source for research on the patient perspective.

When interpreting results or using the current utility values in a CUA, it is also important to consider limitations of vignette-based utility assessment. To maximize accurate representation of patients’ experiences, the health states in this study were carefully drafted based on Internet discussion forum data, iterative steps of clinician input, and detailed literature review. However, health state vignettes are inherently limited by the accuracy of information on which they are based. These limitations are particularly relevant to some of the ART-related risks described in the current health states because published research on these risks is recent, still developing, and not entirely consistent. For example, there is growing consensus that abacavir is associated with elevated cardiovascular risk based on several large observational studies [63–67], but some analyses have not found any statistically significant increase in risk [68–70]. Furthermore, while the bone and renal risks associated with tenofovir disoproxil fumarate have been demonstrated across multiple studies, the impact on most patients seems to involve changes in surrogate markers such as decreased bone mineral density [12–14] and decreased glomerular filtration rate [16, 71–73], often with no associated symptoms. The clinical significance of these laboratory and radiologic changes including the frequency with which these initial problems lead to more serious complications, such as bone fractures and kidney failure, is not yet known.

Therefore, the health states were drafted to reflect this uncertainty of current knowledge so that respondents would not overestimate the impact of the risk. For example, rather than making a definitive statement, the cardiovascular risk health state said “the medication may be associated with a small increase in risk of cardiovascular problems.” Future research with larger samples and longitudinal designs may lead to a better understanding of these risks. If the risks are found to be greater or smaller than currently thought, then the current results could be underestimating or overestimating the disutilities of these risks.

One limitation of the current results is that the disutility of each risk is estimated individually, without considering potential combinations of risks. Some ART regimens may be associated with more than one of these risks, so modelers must decide how best to combine multiple disutility values in cost-utility analyses of treatments with multiple risks. Two or three risks could be combined additively, but this approach may overestimate the disutility of living with risk, thus failing to provide a reasonable estimate of the patient experience. A more conservative approach would be to use the disutility of greatest magnitude when modeling a treatment regimen with multiple risks. For example, to model an ART regimen with risk of both renal problems (disutility = 0.018) and bone problems (disutility = 0.025), the disutility of 0.025 may be used.

Despite limitations, qualitative findings highlight the impact of living with treatment-related risks. In addition, the subsequent TTO valuation study suggests it is feasible to quantify the impact of living with risk in terms of health state utilities. These resulting disutility values for bone, renal, and cardiovascular risk may be useful for differentiating among ARTs in economic modeling of treatment for patients with HIV.

## Appendix: complete health state text

### 1. Basic health state: HIV (this text was also included in all other health states)

- You have a chronic viral infection. There is currently no cure for this infection.
- Without treatment, this virus would cause your immune system to weaken, which could result in life-threatening infections and chronic symptoms (for example, pneumonia, fatigue, unhealthy weight loss, swollen glands, fevers, chills, night sweats, cough).
- You take 1 to 3 tablets every day to keep the infection under control.
- Because of your treatment, your illness is stable and you have no symptoms related to the viral infection. The infection does not interfere with your ability to work or perform daily activities.
- You could spread this virus to other people through sexual contact. However, when you are receiving treatment, your chance of transmitting the virus is significantly reduced.

### 2. ART associated with risk of renal problems

- Your medication is effective for controlling the viral infection.
- However, the medication is associated with a small increase in risk of kidney problems.
- A small number of patients who take this medication will have a decline in kidney function.
- When this happens, there are changes in blood markers that doctors monitor to see how well the kidneys are working.
- These changes in blood markers indicate that the kidneys are not functioning as well as usual. If this persists or worsens, your doctor might change your medication to try to avoid serious kidney problems such as kidney failure.
- Therefore, your kidney function is monitored by your doctor every 3–6 months. This is done with blood and urine tests.

### 3. ART with increased risk of bone problems

- Your medication is effective for controlling the viral infection.
- However, the majority of patients treated with your medication have a small amount of bone mineral density loss. This means that there could be a small decrease in the amount of calcium and other minerals in your bones, which could weaken your bones.
- The decrease in bone mineral density mostly occurs during the first year after starting the medication, but appears to stabilize over time.
- This decrease in bone mineral density likely does not have an impact on most people, but it could lead to a greater risk of bone fractures.
- This treatment-related risk is greater in people who already have other risk factors such as
  - Older age
  - Currently undergoing menopause.
- Personal or family history of bone problems.

### 4. ART associated with risk of cardiovascular events

- Your medication is effective for controlling the viral infection.
- However, the medication may be associated with a small increase in risk of cardiovascular problems, such as a heart attack, which is a sudden blockage in one of the arteries that supplies blood to your heart.
  - A heart attack can cause chest discomfort that feels like heaviness, tightness, and a burning feeling, and treatment usually involves immediate hospitalization.
- Some studies suggest that your current risk of heart attack may double if you take this medication. Other studies have reported a smaller increase in risk.
- This treatment-related risk is greater in people who already have other risk factors such as
  - Older age
  - Diabetes
  - High blood pressure
  - High cholesterol.
- Personal or family history of cardiovascular problems.

### 5. Chronic kidney disease, stage 3 (renal insufficiency)

- Side effect: As a side effect of your medication, you have lost 50% of your kidney function. This means that your kidneys are not cleaning toxins from your blood as well as they used to.
- Symptoms: You do not notice any symptoms related to your kidney function.
- Impact
  - You are able to work and perform your daily routine activities.
  - You are able to socialize, and you can participate in most of your usual physical activities.
- Dietary restrictions
  - To ensure that your condition does not get worse, your doctor recommends that you follow dietary restrictions.
  - You maintain a diet low in potassium and phosphate. Many fruits and vegetables are high in potassium, and phosphate is commonly found in nuts, cheese, and food with preservatives (or pre-prepared food). So you eat less of these foods than you used to.
- Treatment
  - You visit your doctor every 3–4 months. You get a blood test at each visit.
  - You take 1 or 2 tablets up to four times per day.

### 6. Chronic kidney disease, stage 4 (renal insufficiency)

- Side effect: As a side effect of your medication, you have lost 75% of your kidney function. This means that your kidneys are not cleaning toxins from your blood as well as they used to.
- Symptoms
  - Because of this condition, you sometimes experience mild to moderate symptoms such as fatigue, nausea, and lack of appetite.
  - You sometimes feel generally unwell.
- Impact
  - You are usually able to work and perform your daily routine activities. However, there are some days when you do not feel well enough to work.
  - You are usually able to socialize and participate in physical activities, but sometimes you do not feel well enough to do so.
- Dietary restrictions
  - To ensure that your condition does not get worse, your doctor recommends that you follow dietary restrictions.
  - You follow a strict diet that limits you from eating foods high in potassium and phosphate. Many fruits and vegetables are high in potassium, and phosphate is commonly found in nuts, cheese, and food with preservatives (or pre-prepared food). So you have to be very careful about the quantity of these foods that you eat.
- Treatment
  - You visit your doctor every 2–3 months. You get a blood test at each visit.
  - You take 1 or 2 tablets with each meal. You also take additional tablets at other times each day, including the morning and before going to sleep.

### 7. Chronic kidney disease, stage 5 (renal failure with dialysis)

- Side effect: As a side effect of your medication, you have lost 90% of your kidney function. This means that your kidneys are not cleaning toxins from your blood as well as they used to.
- Symptoms
  - Because of this condition, you often experience significant symptoms such as fatigue, nausea, and lack of appetite.
  - You often feel generally unwell.
- Impact
  - You are not able to work. However, you can still perform your daily routine activities.
  - You are very limited in your ability to socialize or participate in physical activities.
- Dietary restrictions
  - To ensure that your condition does not get worse, your doctor recommends that you follow dietary restrictions.
  - You cannot eat foods high in potassium and phosphate. Many fruits and vegetables are high in potassium, and phosphate is commonly found in nuts, cheese, and food with preservatives (or pre-prepared food). So you eat very little of these foods.
- Treatment
  - You visit the hospital 3 times each week for dialysis treatment.
  - In dialysis, you have a tube inserted into a vein (most likely in your neck), which is then attached to a machine that cleans your blood.
  - The dialysis process takes about 4 h, and each visit takes about half of your day.
  - You take 2 to 5 tablets with each meal. You also take additional tablets at other times each day, including the morning and before going to sleep.

### 8. Ongoing effects of prior myocardial infarction

- In the past, you had a heart attack, which is a sudden blockage in one of the arteries that supplies blood to your heart.
- You can participate in regular physical activity.
- You take several medications each day including aspirin, a cholesterol lowering tablet, and a blood pressure lowering tablet.
- You see a doctor at least once every year. At these visits, you receive a range of tests including a blood test and a blood pressure test. At some of these visits, you also receive an ECG (heart rhythm test).
- You have increased responsibility for managing your health. You try to follow your doctor’s recommendations, including
  - Maintaining a healthy weight
  - Exercising regularly
  - Maintaining a low-fat and low-salt diet
  - Limiting stress (e.g., at work)
  - Avoiding smoking.
- Because of your past heart attack, you are at a greater risk for experiencing another heart attack or other cardiovascular problem.
